# Overexpression of an ART1-Interacting Gene *OsNAC016* Improves Al Tolerance in Rice

**DOI:** 10.3390/ijms242317036

**Published:** 2023-12-01

**Authors:** Fuhang Liu, Dan Ma, Jinyu Yu, Ran Meng, Zhigang Wang, Baolei Zhang, Xingxiang Chen, Lin Zhang, Liyun Peng, Jixing Xia

**Affiliations:** State Key Laboratory for Conservation and Utilization of Subtropical Agro-Bioresources, College of Life Science and Technology, Guangxi University, Nanning 530004, China; 1708404002@st.gxu.edu.cn (F.L.); 2208301045@st.gxu.edu.cn (D.M.); 2208301085@st.gxu.edu.cn (J.Y.); 2108301043@st.gxu.edu.cn (R.M.); wangzhigang816@163.com (Z.W.); 1908301074@st.gxu.edu.cn (B.Z.); 1908401001@st.gxu.edu.cn (X.C.); 2108301081@st.gxu.edu.cn (L.Z.); 2008401020@st.gxu.edu.cn (L.P.)

**Keywords:** Al tolerance, ART1, NAC, transcription factor, rice

## Abstract

Rice (*Oryza sativa*) exhibits tremendous aluminum (Al)-tolerance. The C2H2-transcription factor (TF) ART1 critically regulates rice Al tolerance via modulation of specific gene expression. However, little is known about the posttranscriptional ART1 regulation. Here, we identified an ART1-interacted gene *OsNAC016* via a yeast two-hybrid (Y2H) assay. *OsNAC016* was primarily expressed in roots and weakly induced by Al. Immunostaining showed that OsNAC016 was a nuclear protein and localized in all root cells. Knockout of *OsNAC016* did not alter Al sensitivity. Overexpression of *OsNAC016* resulted in less Al aggregation within roots and enhanced Al tolerance in rice. Based on transcriptomic and qRT-PCR evaluations, certain cell-wall-related or ART-regulated gene expressions such as *OsMYB30* and *OsFRDL4* were altered in *OsNAC016*-overexpressing plants. These results indicated that OsNAC016 interacts with ART1 to cooperatively regulate some Al-tolerance genes and is a critical regulatory factor in rice Al tolerance.

## 1. Introduction

Aluminum (Al) is ubiquitously found within soil. In acidic soil, Al takes up an ionic form, which inhibits root growth and mineral absorption, thereby producing dwarf roots with impaired ability to absorb water and nutrients, thus, enhancing plant vulnerability to numerous stressors, including drought [1,2]. Given these factors, Al toxicity substantially restricts crop production in acidic soils.

To remain viable under toxic Al or acidic soil conditions, plants established multiple approaches to internally and/or externally stimulate Al detoxification [3,4]. To internally detoxify Al, plants sequestrate Al within vacuoles, whereby, it is combined with organic acids or other chelators, namely, citrate and oxalate. Alternately, well-established pathways of external Al detoxification involve root-based release of organic acid anions (OAA), including malate, citrate and oxalate release [2,3,4]. These OAAs strongly sequester Al and remove them from the rhizosphere. Recently, the genes encoding malate- and citrate transporters were recognized, for example, Al-activated malate transporter (*ALMT1*) in wheat, and citrate transporters (*HvAACT1* and *SbMATE*) in barley and sorghum [5,6,7].

Rice *Japonica* cultivars demonstrate very high Al tolerance in hydroponic conditions and in soil [8]. Dissimilar to other cereal crops such as barley and wheat, root OAAs release was not one main mechanism for rice Al tolerance. Recent advances in rice Al tolerance research revealed that several kinds of transcription factors (TFs) play critical roles in regulating the expression of Al tolerance genes such as zinc finger protein, WRKY, and MYB [4]. A C2H2 zinc finger TF ART1 (*Al resistance TF1*) is reported to regulate at least 31 down-stream genes that participate in rice Al tolerance [9]. Among them, researchers have functionally characterized ten genes (*STAR1*, *STAR2*, *Nrat1*, *OsALS1*, *OsCDT3*, *OsFRDL2*, *OsFRDL4*, *OsMGT1*, *OsEXPA10*, and *OsART2*) using mutant approach [10,11,12,13,14,15,16,17,18]. For example, *STAR1* and *STAR2* associate to form an ABC transporter, which exports UDP-glucose and critically regulates Al detoxification through cell wall modification [16]. *Nrat1* produces a plasma-membrane-localized transporter protein that transports trivalent Al [10,17], whereas *OsALS1* produces a tonoplast-residing half-size Al-shuttling ABC transporter [18]. *Nrat1* and *OsALS1* work synergistically to internally detoxify Al within rice. On the other hand, *OsFRDL4* encoding a citrate transporter modulates root citrate release, which sequesters Al within the rhizosphere [14]. Moreover, a small cysteine-rich plasma membrane-localized peptide OsCDT3 sequesters Al and blocks its entry into cells [10]. Although the ART1-regulated Al-tolerance gene expressions were enhanced by Al stress, Al did not regulate the mRNA level of *ART1*. Based on these findings, ART1 posttranscriptional or posttranslational modulation might be required for the activation of ART1.

The plant-specific NAC (NAM, ATAF, CUC1/2) TF family dominates the TF population within plants [19]. At present, we recognize 117 Arabidopsis and 151 rice NAC proteins [19]. Generally, NAC proteins contain a conserved target-interacting domain (NAC domain) at the N-terminal region and a highly diverse transcriptional regulating C-terminal domain. NAC TFs are critical regulators of various stress responses such as cold, salt, drought and ABA stresses [20,21]. Recently, several studies have reported the involvement of NAC TFs in Al stress response within plants. Rice bean NAC TF VuNAR1, for example, critically regulates Al tolerance through the regulation of cell wall pectin metabolism in Arabidopsis [22]. A NAC TF SOG1 (GAMMA RESPONSE1 SUPPRESSOR) plays a central role in DNA damage response. Disruption of SOG1 showed hypersensitivity to high Al treatment [23]. More recently, ANAC017 was reported to participate in Al tolerance via modulation of xyloglucan transglucosylase/hydrolase 31 (XTH31) expression in Arabidopsis [24]. However, the significance of NAC TF in regulating rice Al tolerance remains unknown.

To gain insights into how ART1 is regulated at the posttranscriptional level, we screened for ART1-interacting proteins via yeast two-hybrid (Y2H) assay with truncated ART1 as bait and identified a NAC TF OsNAC016. We investigated the phenotypes of *OsNAC016*-knockout and overexpressing lines under normal and Al treatment conditions. We also analyzed the root transcriptomes of *OsNAC016*-overexpressing and wild-type (WT) plants with or without Al treatment. The presented evidence revealed that OsNAC016 is involved in rice Al tolerance via the modulation of certain cell-wall-related genes and partial ART1-regulated genes.

## 2. Results

### 2.1. Identification of ART1-Interacting Protein OsNAC016

**Figure 1:**
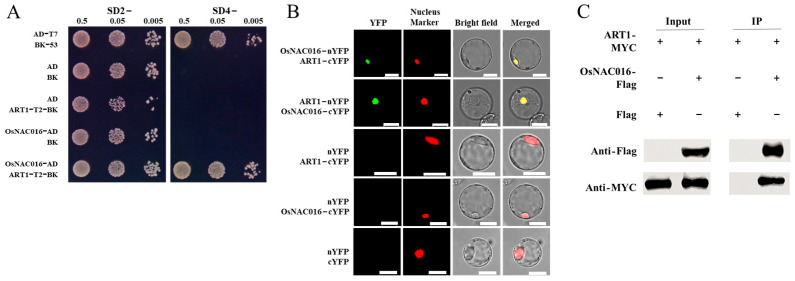
Interaction of OsNAC016 with ART1. (**A**) Interaction between OsNAC016 and truncated ART1 examined by Y2H assay. SD2− medium lacking leucine and tryptophan, SD4− medium lacking tryptophan, leucine, histidine, and adenine. (**B**) OsNAC016-ART1 interaction shown by a BiFC assay. Pair of plasmid constructs (positive or negative control, *OsNAC016-nYFP* and *ART1-cYFP*, *ART1-nYFP* and *OsNAC016-cYFP*) with Ghd7-RFP were transiently co-expressed in rice protoplasmic cells. YFP (green), RFP (red), and merged image (yellow) of YFP (green) and RFP (red) were shown. Ghd7-RFP was used as a nuclear marker. Bar = 10 μm. (**C**) Coimmunoprecipitation of OsNAC016 with ART1. *OsNAC016-Flag* and *ART1-MYC* were transiently co-expressed in *N. benthamiana* leaves. Crude proteins were immunoprecipitated using anti-Flag antibody-conjugated agarose beads and then detected using anti-Flag and anti-MYC antibodies, respectively.

A previous study showed that ART1 can transcriptionally activate target genes within yeast. Truncation analysis indicated that its activation domain resides within its C terminus (176–466 aa). To probe the molecular mechanism of ART1 activation by Al, we utilized truncated ART1 (1–175 aa) lacking an activation domain as bait to screen for ART1-interacting proteins via yeast two-hybrid (Y2H) assay and identified eight candidate proteins (Figure 1A; Supplemental Figure S1A,B; Table S1). As several NAC TFs were reported to be involved in regulating Al tolerance in plants [22,23], we selected a NAC TF OsNAC016 (LOC_Os01g01430) for further functional analysis. *OsNAC016* encodes a 340-amino-acid protein harboring an NAM domain at its N terminus. To verify a physical association between OsNAC016 and ART1, we conducted a bimolecular fluorescence complementation (BiFC) assessment in rice protoplast cells. We observed the YFP signal in rice protoplasts co-expressing OsNAC016-nYFP and ART1-cYFP or ART1-nYFP and OsNAC016-cYFP, but not in those co-expressing control constructs. Furthermore, YFP signal nicely overlapped with the RFP signal of the nuclear marker Ghd7 [25] (Figure 1B), confirming in vivo interaction of OsNAC016 and ART1 at the nucleus. We also performed co-immunoprecipitation (Co-IP) experiment in *N. benthamiana* leaves co-expressing MYC-tagged OsNAC016 and FLAG-tagged ART1 and found that OsNAC016 was coimmunoprecipitated with ART1 in planta (Figure 1C). Taken together, the aforementioned evidence indicated the binding of OsNAC016 to ART1 within the nuclear compartment of plant cells.

### 2.2. Expression Pattern of OsNAC016

**Figure 2:**
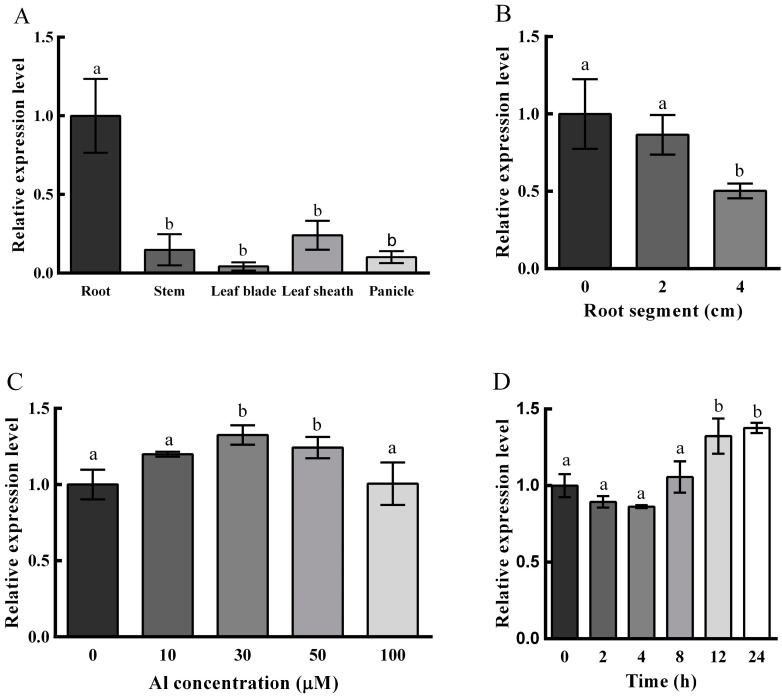
Expression pattern of *OsNAC016*. (**A**) Expression of *OsNAC016* in various tissues of rice cv. Nipponbare (WT). (**B**) Expression in different root segments. RNA was extracted from different root segments (0–1, 1–2, and 2–3 cm from root apex) of 5-d-old wild-type rice seedlings. (**C**) Dose-dependent expression of *OsNAC016* in rice roots with Al treatment. Seedlings were exposed to different concentrations of Al at pH 4.5 for 8 h. (**D**) Time-dependent expression of *OsNAC016* in rice roots with Al treatment. Seedlings were treated with 30 μM Al at pH 4.5 for different times. *Histone H3* was used as an internal standard. Data are means ± SD of three biological replicates. Significant differences were determined by Tukey’s test and are labeled with different letters (*p* < 0.05).

QRT-PCR assessment was utilized to determine the *OsNAC016* expression within roots, stems, leaf blades, leaf sheaths and panicles. OsNAC016 was primarily expressed in roots (Figure 2A), and its expression was higher in the root tip (0–1 cm) than in the mature root segments (1–2 and 2–3 cm) (Figure 2B). Dose-reliant and time course assessments revealed that the root OsNAC016 expression was weakly up-regulated by Al treatments (Figure 2C,D).

### 2.3. Subcellular and Cellular Localization of OsNAC016

**Figure 3:**
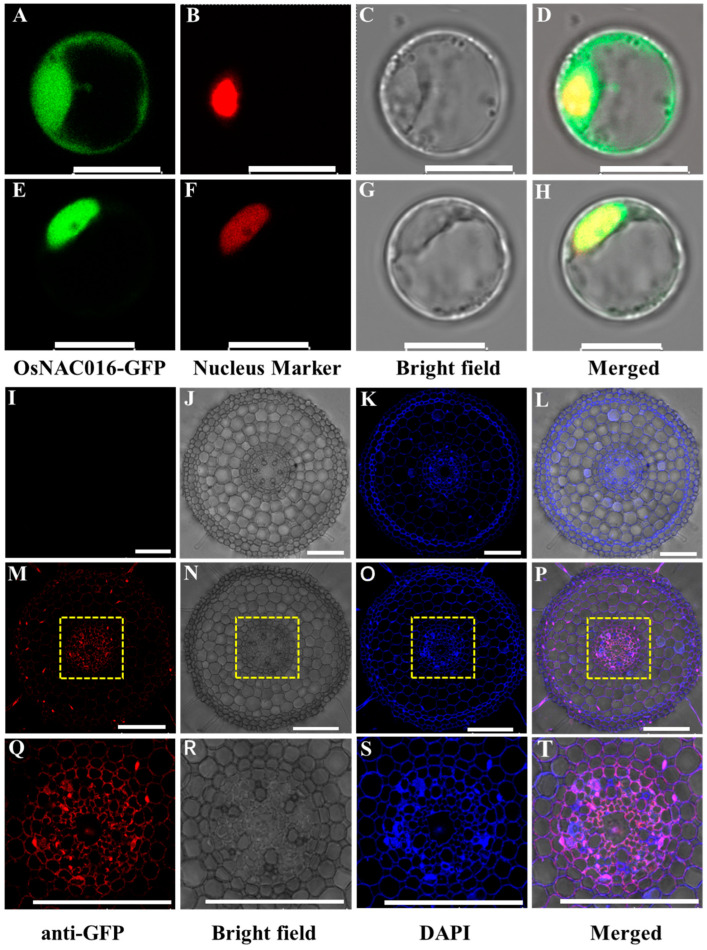
Subcellular and cellular localization of OsNAC016. (**A**–**H**) Subcellular localization of OsNAC016. *GFP* (**A**–**D**) or *OsNAC016-GFP* (**E**–**H**) plus *Ghd7-RFP* was separately transformed into rice protoplast cells. GFP (green), RFP (red), and merged image (yellow) of YFP (green) and RFP (red) were shown. Bars = 10 μm. (**I**–**T**) Cellular localization of OsNAC016. Immunostaining of the roots of Nipponbare (**I**–**L**) and *ProOsNAC016:OsNAC016-GFP* transgenic plants (**M**–**P**) was performed using the anti-GFP antibody. (**Q**–**T**) Magnified images of the yellow boxed areas in (**M**–**P**), respectively. Red shows the fluorescent signal of the anti-GFP antibody, blue shows autofluorescence of cell wall and nuclei stained with DAPI. Scale bar = 100 μm.

To investigate the OsNAC016 subcellular localization, the OsNAC016-GFP fusion construct or GFP empty vector was co-introduced with the nuclear marker Ghd7-RFP into rice protoplast cells. The resulting OsNAC016-GFP fluorescence showed a perfect overlap with the red fluorescence produced by Ghd7-RFP while the green fluorescence of GFP alone were widely distributed in the nucleus and cytoplasm. Based on this evidence, OsNAC016 primarily resides in the nucleus (Figure 3A–H).

To determine the OsNAC016 cell-specific localization in rice roots, we conducted immunostaining of the transgenic rice expressing the *ProOsNAC016:OsNAC016-GFP* construct. GFP antibody co-staining with a nuclear stain DAPI revealed that red fluorescence was merged with the blue DAPI fluorescence in all the root cell layers (Figure 3M–T). No red fluorescence was evident in the WT root (Figure 3I–L). These results indicated that OsNAC016 locates to the nucleus of all root cells.

### 2.4. Mutation of OsNAC016 Did Not Affect Al Tolerance in Rice

**Figure 4:**
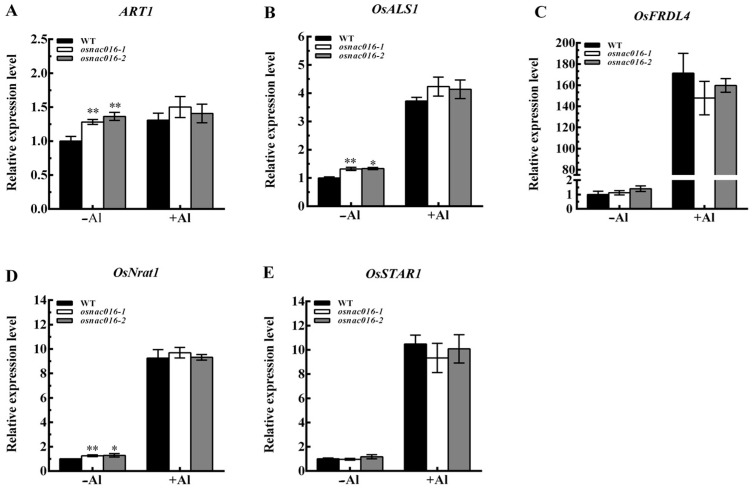
Effect of knockout of *OsNAC016* on the expression of *ART1* and some ART1-regulated genes in roots. *ART1* (**A**), *OsALS1* (**B**), *OsFRDL4* (**C**), *OsNrat1* (**D**), *OsSTAR1* (**E**). Both wild-type rice and two *OsNAC016*-knockout lines were exposed to 0 or 50 µM Al for 8 h. *Histone H3* was used as an internal standard. Data are means ± SD (n = 3). Asterisks indicate significant differences between WT and the knockout lines (Tukey’s test, ** *p* < 0.01, * *p* < 0.05).

**Figure 5:**
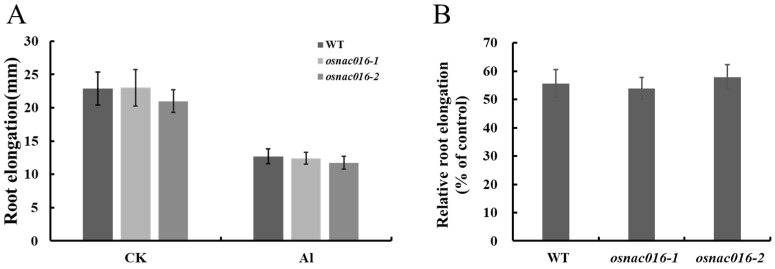
Sensitivity analysis of *OsNAC016*-knockout lines to Al. (**A**) Effect of Al on root length. (**B**) Effect of Al on root elongation. Five-day-old seedlings of the WT and two *OsNAC016*-knockout lines were exposed to a 0.5 mM CaCl_2_ solution, pH 4.5, containing 0 or 50 μM Al for 24 h. Root length was measured before and after the treatment. Relative root elongation refers to (root elongation with Al)/(root elongation without Al) × 100. Data are means ± SD (n = 8).

To test whether *OsNAC016* participated in rice Al-tolerance, we created two null mutants for *OsNAC016* via CRISPR/Cas9-mediated gene editing; one with a 1-bp insertion (*osnac016-1*) and another with a 307-bp deletion (*osnac016-2*), at varying sites within the first exon (Supplemental Figure S2). Additionally, the expression level of *OsNAC016* was significantly reduced in the two *osnac016* lines compared with WT (Supplemental Figure S3). First, we compared the *ART1* and some ART1-regulated gene expressions in roots between WT and two *OsNAC016*-knockout rice lines with or without Al treatment. Under Al-free conditions, the *ART1*, *OsASL1* and *OsNrat1* expressions were slightly elevated in the *OsNAC016*-knockout lines, relative to the WT rice (Figure 4A,B,D). However, following Al exposure, the gene expressions were similar between WT rice and two *OsNAC016*-knockout lines (Figure 4A–E). Next, we evaluated WT rice and two *OsNAC016*-knockout line root growth, and demonstrated similar root growth in the two *osnac016* mutants and WT rice both with and without Al (Figure 5A,B). Additionally, phylogenetic analysis showed that multiple homologs for OsNAC016 exist in the rice genome such as OsNAC103, OsNAC58, OsNAC122, and OsNAC131 (Supplemental Figure S4). Like *OsNAC016*, the expression levels of *OsNAC58*, *OsNAC103*, *OsNAC122*, and *OsNAC131* in roots were weakly affected by Al treatment (Supplemental Figure S5). The aforementioned results indicated that no differences in *ART1* and some ART1-regulated gene expressions as well as Al tolerance between the WT and mutant lines under Al stress might be explained by the emergence of redundant genes with comparable activities to OsNAC016 in rice.

### 2.5. Overexpression of OsNAC016 Improves Al Tolerance in Rice

**Figure 6:**
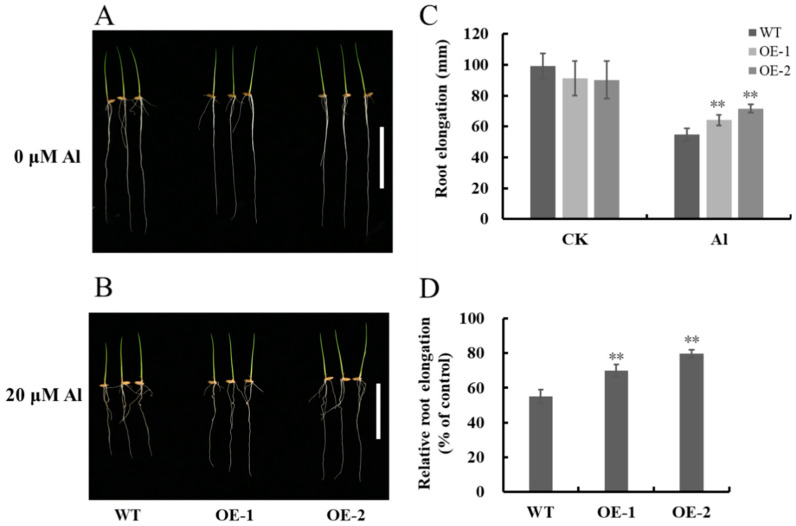
Al sensitivity of the WT and *OsNAC016*-overexpressing lines. (**A**,**B**) Phenotypes of the WT and *OsNAC016*-overexpressing lines after the treatment with 0 (**A**) and 20 μM Al (**B**). (**C**) Effect of Al on root length. (**D**) Effect of Al on root elongation. Germinated seeds of each line were exposed to a 0.5 mM CaCl_2_ solution (pH 4.5) containing 0 or 20 μM Al for 5 d. Scale bar = 5 cm. Root length was measured before and after the treatment. Relative root elongation refers to (root elongation with Al)/(root elongation without Al) × 100. Data are means ± SD of biological replication (n = 8). Asterisks indicates significant differences (*p* < 0.01, Tukey’s test).

To further elucidate the *OsNAC016*-mediated regulation of rice Al-tolerance, we generated *OsNAC016*-overexpressing lines in which *OsNAC016* is regulated by the maize *ubiquitin 1* promoter. The root *OsNAC016* expression within two independent transgenic lines was increased by more than 70 times (Supplemental Figure S6). Under Al-free conditions, root growth of the two *OsNAC016*-overexpressing line was comparable to WT rice. Alternately, with 20 μM Al exposure, WT root elongation was strongly suppressed, compared to the two overexpression lines of *OsNAC016* (Figure 6A–D). Therefore, *OsNAC016* overexpression can enhance Al tolerance in rice.

**Figure 7:**
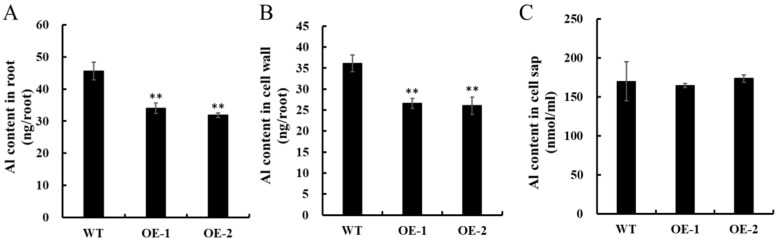
Al accumulation in the root tips. (**A**) Al content in the root tips. (**B**) Al content in the cell wall of root tips. (**C**) Al concentration in the cell sap of root tips. Seedlings (5 days old) of the WT and two *OsNAC016*-overexpressing lines (OE-1, OE-2) were exposed to a solution (0.5 mM CaCl_2_, 50 μM AlCl_3_, pH = 4.5) for 8 h. Root tips (0–1 cm) were excised and Al content in the root tips, cell wall, and cell sap was measured by ICP-MS. Data were means ± SD (n = 3). Asterisks indicates significant differences (*p* < 0.01, Tukey’s test).

Moreover, we compared the root Al levels within WT rice and two *OsNAC016*-overexpressing lines. After a short-term (8 h) treatment with 50 μM Al, Al levels within roots and the cell walls within root tips (0 to 1 cm from the apex) were drastically reduced in the overexpression lines, relative to the WT (Figure 7A,B). However, Al concentration within root cell sap among all lines was comparable (Figure 7C). Collectively, this suggested that *OsNAC016* overexpression reduces Al accumulation within root tip cell walls.

### 2.6. Overexpression of OsNAC016 Modulates Multiple Gene Expressions in Rice

**Figure 8:**
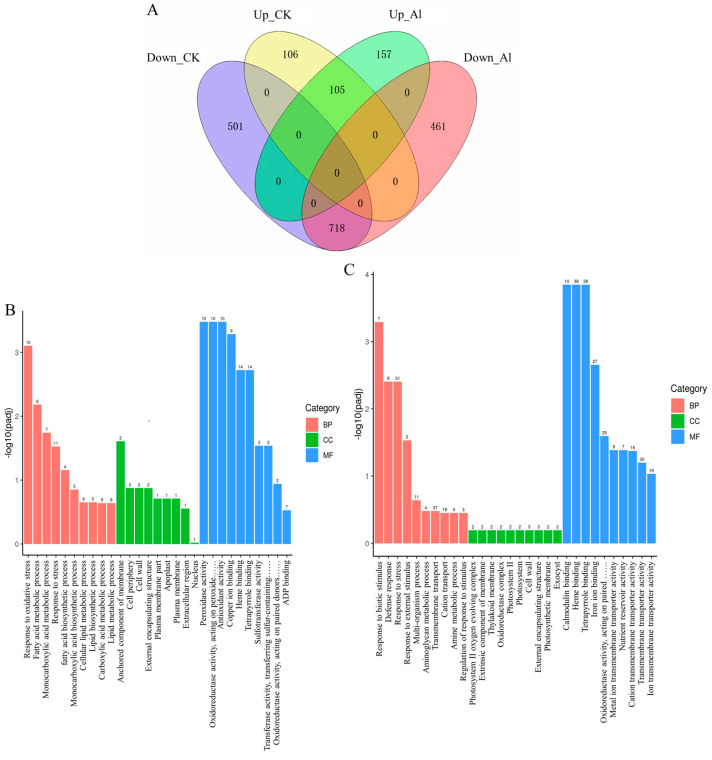
Transcriptome analysis of OsNAC016-regulated genes. (**A**): Venn diagram of the numbers of OsNAC016-regulated genes in the OE and WT lines. UP_CK: the up-regulated genes in the OE compared with the WT under normal conditions (fold-change > 2). UP_Al: the up-regulated genes in the OE compared with the WT under Al treatments (fold-change > 2). DOWN_Al: the down-regulated genes in the OE compared with the WT under Al treatments (fold-change < 0.5). DOWN_CK: the down-regulated genes in the OE compared with the WT under normal conditions (fold-change < 0.5). (**B**,**C**) Classification of up-regulated genes (**B**) and down-regulated genes (**C**) in OE line compared with the WT under Al treatments. BP: biological process. CC: cellular component. MF: molecular function. Number represents the numbers of the genes. Three biological replicates (n = 3) were performed for each treatment.

**Figure 9:**
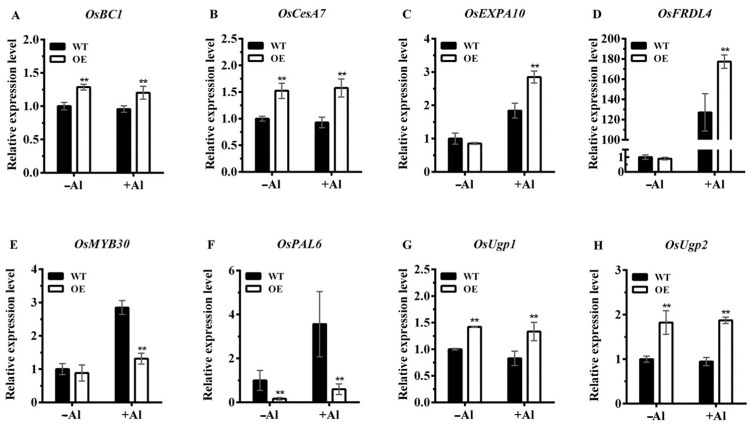
The expression level of eight candidate DEGs in WT and OE (*OsNAC016* overexpression) roots under normal conditions and Al treatments. *OsBC1* (**A**), *OsCesA7* (**B**), *OsEXPA10* (**C**), *OsFRDL4* (**D**), *OsMYB30* (**E**), *OsPAL6* (**F**), *OsUgp1* (**G**), *OsUgp2* (**H**). −Al represents WT and OE lines under normal conditions. +Al represents WT and OE lines under Al treatment conditions (0.5 mM CaCl_2_, 40 μM AlCl_3_, pH = 4.5) for 8 h. *Histone H3* was used as an internal standard. Data are means ± SD (n = 3). Asterisks indicates significant differences (*p* < 0.01, Tukey’s test).

To help understand the possible mechanisms of the enhanced Al tolerance, we analyzed the transcriptomes of Nipponbare (WT) and *OsNAC016*-overexpressed line (OE-1) rice roots treated with 0 or 40 μM Al for 8 h via an RNA-seq approach. Based on a threshold of twofold change, under normal conditions, we identified 1430 differentially expressed genes (DEGs) between the WT and OE line, and following Al exposure, 1441 DEGs between the lines (Figure 8A; Table S2). Among them, there were 718 down-expressed DEGs and 105 up-expressed DEGs under both two conditions (Table S2). These 823 genes were considered as the OsNAC016-regulated genes under Al-treated conditions. Based on gene ontology (GO) enrichment analysis, the up-expressed genes were primarily related to oxidative stress response and fatty acid metabolic process (Figure 8B), while the down-expressed genes were primarily involved in stress response and transmembrane transport (Figure 8C). In the cellular component category, some up- and down-regulated genes were enriched in the cell wall group. To further validate the RNA-seq data reliability, we chose seven stress- or cell-wall-related genes for RT-qPCR analysis, including *OsPAL6* (Os04g0518400), *OsEXPA10* (Os04g0583500), *OsMYB30* (Os02g0624300), *OsCesA7* (Os10g0467800), *OsBC1* (Os03g0416200), *OsUgp1* (Os09g0553200) and *OsUgp2* (Os02g0117700). Our RT-qPCR results corroborated our RNA-seq information, confirming the RNA-seq data reliability (Figure 9A–H). Therefore, based on these findings, OsNAC016 likely modulated both stress- and cell-wall-associated genes to induce Al stress resistance within rice.

Since OsNAC016 interacted with ART1, overexpression of *OsNAC016* might influence the ART1-modulated gene expressions. To verify this hypothesis, we assessed the expressions of 31 ART1-regulated downstream genes between WT and OE line in RNA-seq data (Supplemental Table S3). Among these genes, most genes were similarly upregulated by Al treatment between two lines. However, four genes (Os02g0770800, Os01g0919200, Os01g0919100, Os01g0731600) showed largely elevated expression differences between OE line versus WT rice. Among them, only Os01g0919100 (OsFLRD4) was functionally characterized and was involved in rice Al tolerance. We conducted RT-qPCR analysis and confirmed that the *OsFLRD4* expression was substantially elevated in the *OsNAC016*-overexpressing lines, relative to WT rice (Figure 9D). Therefore, *OsNAC016* overexpression strongly modulates the expressions of partial ART1-regulated genes.

## 3. Discussion

In this study, we obtained an ART1-interacted gene *OsNAC016* using a yeast two-hybrid (Y2H) assay. A physical association between ART1 and OsNAC016 was further proved via BiFC and Co-IP experiments. *OsNAC016* was primarily expressed within roots and slightly modulated via Al. Immunostaining showed that OsNAC016 was a nuclear protein and localized in all root cells. Knockout of *OsNAC016* has a weak or no effect on the expressions of *ART1* and ART1-modulated Al-tolerant genes under normal and Al stress. Furthermore, we observed no marked alterations in Al tolerance between WT and *osnac016* mutants. One possibility is that there are redundant genes with similar function to OsNAC016, leading to no obvious Al sensitive phenotype in *osnac016* mutants. This notion is supported by the presence of multiple homologs for OsNAC016 in the rice genome such as OsNAC58, OsNAC103, OsNAC122, and OsNAC131. On the other hand, overexpression of *OsNAC016* resulted in less Al root aggregation and enhanced rice tolerance to Al stress. Based on transcriptomic sequencing and qRT-PCR assessment, the expressions of some genes related to cell wall or Al stress were altered in *OsNAC016*-overexpressing plants. These findings suggested that OsNAC016 contributes to the regulation of Al tolerance within rice.

The cell wall acts as an initial physical barrier that protects plant cells from harmful external environmental factors, such as, Al toxicity. Among the main Al-resistance strategies in plants is to decrease Al accumulation in the cell wall through modifying cell wall properties to alter its Al-binding capacity [26,27,28,29]. Normally, high Al tolerance in plants is associated with low Al accumulation in the cell wall, as described in rice [27], *Secale sylvestre* [29], and *Arabidopsis* [28]. Interestingly, *OsNAC016* overexpressing lines showed reduced cell wall Al content and enhanced Al tolerance relative to the WT rice. The RNA-seq and qRT-PCR analyses demonstrated that the expression levels of some genes related to cell wall modification in the *OsNAC016*-overexpressing lines were significantly downregulated or upregulated relative to the WT rice under Al stress, including *OsPAL6*, *OsMYB30*, *OsEXPA10*, *OsBC1*, *OsCesA7*, *OsUgp1* and *OsUgp2.* Among them, *OsPAL6* and *OsMYB30* are down-regulated while the other five genes are up-regulated in the *OsNAC016*-overexpressing lines. OsPAL6, a phenylalanine ammonia-lyase, forms the first major enzyme in the phenylpropanoid pathway, participating in lignin biosynthesis [30]. Several studies have showed that down-regulation or knockout of lignin biosynthesis-related genes impact the Al interaction with the cell wall and Al tolerance such as *4-coumarate: coenzyme A ligase 4CL4* and *4CL5* within rice [26,31]. The expression of *OsPAL6* and *4CL5* is positively regulated by OsMYB30 [26,30,31]. Knockout of *OsMYB30* resulted in a lower cell wall Al aggregation and an enhanced Al tolerance within rice [26]. *OsEXPA10* encoding a cell wall expansin participated in root cell elongation through the modulation of cell-wall loosening. Knocking out *OsEXPA10* led to a reduction in root length [11]. OsBC1 belonging to the classical dynamin-related protein family (OsDRPs), is homologous to OsBC3/OsDRP2B. Disruption of *OsBC3* caused a marked rise in cell wall pectin content [32]. Generally, pectic matrix is proposed to be the primary cell wall Al-binding site [33]. OsCesA7 is cellulose synthase A subunit 7 functioning in cellulose biosynthesis. Mutation of *OsCesA7* resulted in a reduced cellulose and an increased cell wall hemicellulose content [34]. Hemicellulose possesses Al-binding ability. Its content in the cell wall affects Al accumulation and tolerance [31]. Both *OsUgp1* and *OsUgp2* encode UDP-glucose pyrophosphorylase (UGPase), which accelerates the UTP and glucose-1-phosphate conversion to pyrophosphate and UDP-glucose [35]. UDP-glucose is reported to be used to modify the cell wall to alleviate Al stress-induced root growth inhibition through masking cell-wall-based Al-binding sites. Therefore, the reduced cell wall Al aggregation in roots found within *OsNAC016*-overexpressing lines may be explained via modulation of the above-mentioned gene expressions, relative to the WT rice.

ART1 is the central regulator for rice Al tolerance, which regulate ≥31 Al-detoxifying genes. Overexpression of *OsNAC016* significantly increases the partial ART1-modulated gene expressions such as *OsFRDL4*, Os02g0770800, Os01g0919200, and Os01g0731600. Among the previously characterized ART1-modulated genes, *OsFRDL4* expression was the most affected. OsFRDL4 is a plasma-membrane-localized citrate transporter that facilitates rice Al tolerance through releasing citrate from roots following Al stimuli. ART1 regulates *OsFRDL4* expression by directly interacting with its promoter’s cis-element GGN(T/g/a/C)V(C/A/g)S(C/G) region [14]. A recent study showed that OsNAC016 can bind to the cis-element CACG-motif [36]. We performed an analysis of cis-acting elements and identified 11 CACG-motifs in the 1–1642 bp promoter region of *OsFRDL4* (Supplemental Figure S7). Some OsNAC016-binding motifs are adjacent to ART1-interacting cis-element. These suggested that overexpression of *OsNAC016* might cause more OsNAC016-ART1 complexes to interact with the *OsFRDL4* promoter region to promote its expression and citrate secretion under Al stress conditions, resulting in an enhanced Al tolerance within rice.

Additionally, transcriptome analysis revealed that overexpression of *OsNAC016* up-regulated many biological processes-related gene expressions such as peroxidase activity, antioxidant activity, and response to oxidative stress under normal conditions, implying that OsNAC016 may also play important regulatory roles in other stress responses. A recent study reported that OsNAC016 negatively regulated drought tolerance via down-regulating ABA- and drought-responsive genes in rice [37]. More recently, OsNAC016 was also shown to negatively modulate Pi-starvation tolerance though the activation of *OsSPX2* in rice [36]. These findings suggested that OsNAC016 acts as an important TF that regulates multiple stress responses in rice.

In conclusion, OsNAC016 is the first protein identified as ART1-interacting protein. Overexpression of *OsNAC016* can lower root cell wall Al aggregation through modulation of some cell-wall-related and ART1-modulated gene expressions and thus improve Al tolerance in rice.

## 4. Materials and Methods

### 4.1. Plant Materials and Growth Conditions

Herein, we used WT (*Oryza sativa* cv Nipponbare), as well as 2 *OsNAC016*-knockout lines, and 2 *OsNAC016* overexpression lines which were generated within the laboratory. Rice seeds underwent a 2-day submersion in deionized water without light at 28 °C. Germinated seeds were transferred to a floating net under a solution with 0.5 mM CaCl_2_ in an illumination incubator and used for various experiments.

Roots, stems, leaf blades, leaf sheaths and panicles were extracted from WT plants at the heading stage for organ-specific expression analysis of *OsNAC016*. We examined the spatial *OsNAC016* expression by individually harvesting root tips (0–1 cm), basal roots (1–2 cm) and mature region (2–3 cm). To analyze the *OsNAC016*-mediated response to Al stress, rice seedlings (5 days old) were treated to varying Al concentrations (0, 10, 30, 50, 100 μM) at pH 4.5 for 8 h or 30 μM Al over a wide range of experiment durations (0, 2, 4, 8, 12 and 24 h).

### 4.2. Analysis of Al Tolerance

For short-term Al treatment, seedlings (5-d-old) from individual lines underwent a 24-h treatment with a 0.5 mM CaCl_2_ and 50 μM Al solution (pH 4.5). For long-term Al treatment, seedlings (2-d-old) from individual lines underwent a 5-d treatment with a 0.5 mM CaCl_2_ and 20 μM Al solution (pH 4.5). Root lengths were recorded both prior to and after treatments using a ruler. Relative root elongation was computed using the following formular: (Al-treated root elongation)/(Al-untreated root elongation) × 100.

### 4.3. Generation of Transgenic Rice Plants

To construct the overexpression vector for *OsNAC016*, *OsNAC016* cDNA was amplified from the Nipponbare root cDNA via PCR and specific primers. The amplified cDNA fragment was ligated to the pCAMBIA1300-Ubi vector between the maize *Ubiquitin 1* promoter and nopaline synthase terminator, generating the pCAMBIA1300-*Ubi1:OsNAC016* construct. To create the CRISPR construct for *OsNAC016*, two sgRNA expression cassettes containing two different specific target sites for OsNAC016 were digested by *Bsa*I and cloned into a pYLCRISPR/Cas9 vector [38], producing the pYLCRISPR-*OsNAC016* construct. These constructs were next introduced into rice cv. Nipponbare via *Agrobacterium tumefaciens* strain EHA105-mediated transformation. The employed primer sequences are summarized in Table S4.

### 4.4. RNA Extraction and Gene Expression Analysis

The Trizol reagent kit (Invitrogen, Carlsbad, CA, USA) was utilized for total RNA extraction as per kit directions. The Hiscript II Q RT SuperMix Kit (Vazyme, Nanjing, China) was then employed to eliminate genomic DNA and perform reverse transcription reactions. For qRT-PCR, ChanQTM SYBR Color qPCR Master Mix (Vazyme, Nanjing, China) was used in a StepOnePlus RT-PCR System (Analytik Jena AG). An internal standard of *Histone H3* was utilized. Relative gene expression employed the 2^−ΔΔCT^ calculation method. The primers for qRT-PCR were shown in Table S4.

### 4.5. OsNAC016 Subcellular Localization

To assess OsNAC016 intracellular distribution, we inserted the coding sequence of *OsNAC016* into the pYL322-GFP vector upstream of GFP to create the pYL322-*OsNAC016-GFP* construct. Rice protoplasts were co-transfected with either pYL322-*OsNAC016-GFP* or a control vector along with a nuclear marker (OsGhd7-RFP), following the protocols described by Chen et al. [39]. After 12–16 h incubation, GFP or RFP fluorescence was observed using a confocal laser scanning microscope (TCS SP8; Leica, Weztlar, Germany).

### 4.6. OsNAC016 Cellular Distribution

The promoter (2000 bp) and CDS (1023 bp) sequences of *OsNAC016* were amplified using specific primers Pro-NAC016-F/Pro-NAC016-R and 1300-NAC016-F/1300-NAC016-R, respectively. Subsequently, the promoter and CDS fragments were cloned into the *Hind*III and *BamH*I sites of the pCAMBIA1300-*GFP* vector. The subsequent construct (pCAMBIA1300-*ProOsNAC016:OsNAC016-GFP*) was introduced into Nipponbare to generate the transgenic lines harboring *ProOsNAC016-OsNAC016-GFP*. The employed primer sequences are summarized in Supplemental Table S4.

To examine the OsNAC016 cellular localization, immunostaining was carried out using a GFP antibody following the protocol described by Yamaji and Ma in 2007 [40]. In brief, roots from both WT (WT) plants and transgenic lines expressing *ProOsNAC016:OsNAC016-GFP* were embedded in a 5% agarose and sliced into sections (100-μm thickness) using a microslicer (VT1000 S Leica), which were then transferred to microscope slides for incubation with rabbit anti-GFP polyclonal antibodies, with subsequent incubation with secondary antibodies (Alexa Fluor 555 goat anti-rabbit IgG; Molecular Probes) at room temperature (RT). Cell nucleus was stained with DAPI. Fluorescent images were then taken via a confocal laser scanning microscope (Leica model TCS SP8, Weztlar, Germany).

### 4.7. Y2H Assy

We screened a yeast cDNA library, prepared from the 12-h 30 µM Al-treated roots of 5-day-old WT rice seedling using truncated ART1 CDS (1–525 bp) as bait and the Matchmaker Yeast Two-Hybrid System (Clontech, Shiga, Japan). To verify the truncated ART1 and target protein associations, the truncated ART1 and target gene coding sequences were inserted into pGBKT7 and pGADT7 vectors, respectively. The resulting bait and prey vectors were co-introduced into the yeast strain AH109 and cultured on an SD medium without Leu and Trp or lacking Leu, Trp, Ade, and His. The employed primer sequences are summarized in Supplemental Data.

### 4.8. BiFC Assay

To prepare the BiFC constructs, the *OsNAC016* and *ART1* coding sequences were amplified from the Nipponbare root cDNA using corresponding primers, prior to cloning. The amplified products were cloned into the BiFC vectors pUC19-35S-Vn and pUC19-35S-Vc, producing the OsNAC016-cYFP, OsNAC016-nYFP, OsART1-cYFP and ART1-nYFP constructs. We prepared rice protoplast and conducted plasmid transformation into cells as reported previously [39]. Following plasmid transformation, cells were maintained without light at 28 °C for 12–15 h. Fluorescent images were captured via confocal laser scanning microscope (TCS SP8; Leica, Weztlar, Germany). The employed primer sequences are summarized in Table S4.

### 4.9. Co-IP Assay

The *OsNAC016* and *ART1* full-length coding sequences were ligated into the *Kpn*I and *BamH*I sites of pCAMBIA1300-35S-FLAG and pCAMBIA1300-35S-MYC vectors, respectively. The pCAMBIA1300-*35S:OsNAC016-FLAG* and pCAMBIA1300-*35S:ART1-MYC* recombinant constructs were introduced into *Agrobacterium* strain (GV3101), prior to transformation into 4-week-old *N. benthamiana* leaves. Approximately 24 h later, total protein isolation was completed from infected leaves by Lysis extract NB1 buffer (50 mM Tris-MES [pH 7.3], 500 mM Sucrose, 1 mM MgCl_2_, 10 mM EDTA, 5 mM DTT, 1 mM PMSF, 100× Cocktail). Protein co-IP experiment was performed as reported by Xu et al. (2015) [41]. Proteins were detected with anti-FLAG (M2008S, Abmart, Shanghai, China, 1:5000) and anti-MYC (M2002S, Abmart, Shanghai, China, 1:5000) antibodies.

### 4.10. Evaluation of Root Tip Al Content

Five-day-old WT and two *OsNAC016*-overexpressing line seedlings were exposed for 8 h to a solution (pH 4.5) with 0.5 mM CaCl_2_ and 50 µM AlCl_3_. After 3 rinses in 0.5 mM CaCl_2_, 20 root segments (0–1 cm from the root tip) each replicate were extracted and then transferred to a 2 mL tube or ultrafree-MC centrifugal filter unit (Millipore, Billerica, MA, USA). To obtain root cell sap, we centrifuged root-containing units for 10 min at 3000× *g* at 4 °C. After that, the units were frozen overnight at −80 °C. The root cell sap solution was obtained via centrifugation of samples at 20,400× *g* for 10 min at RT. The residues in the units were washed three times with alcohol (70%) by a vortex. The cell sap was diluted with 5% nitric acid. Both cell walls and whole roots were digested by 65% nitric acid. The Al contents of cell sap, cell wall and whole root tip were assessed using ICP-MS (Plasma Quant MS; Analytik Jena AG).

### 4.11. RNA-Seq Assay

Five-day-old WT and *OsNAC016*-overexpression line (OE) seedlings were subjected to a 0.5 mM CaCl_2_ solution (pH 4.5) with 0 or 50 µM AlCl_3_ for 8 h. The seedling roots were sampled for RNA-seq experiments. Following total root RNA extraction, we synthesized corresponding cDNA, and conducted cDNA library generation. cDNA library sequencing was conducted with the Illumina NovaSeq 6000 platform. Deseq2 was employed for gene expression comparison analysis between WT and OE lines. DEGs were identified using the following criteria: |log2(FoldChange)| ≥ 1 and padj ≤ 0.05. DEGs GO (Gene Ontology; http://geneontology.org/, accessed on 1 October 2022) analysis was done using hypergeometric tests, and *p*-values were utilized for determining significant enrichment of corresponding categories.

## Data Availability

All the data supporting the conclusions of this article are provided within the article and in its additional files. All data and materials are available upon reasonable request from the corresponding author.

## Supplementary Materials

The following supporting information can be downloaded at: https://www.mdpi.com/article/10.3390/ijms242317036/s1.
