# Supplementary material for: Overexpression of an ART1-Interacting Gene OsNAC016 Improves Al Tolerance in Rice
Source: Int J Mol Sci. 2023 Dec 1;24(23):17036. doi: 10.3390/ijms242317036 (PMC10706868; doi:10.3390/ijms242317036)
Supplement: Supplementary file 1 [file ijms-24-17036-s001.zip › Supplemental Figure S1-S7.pdf]

Supplemental Figure.

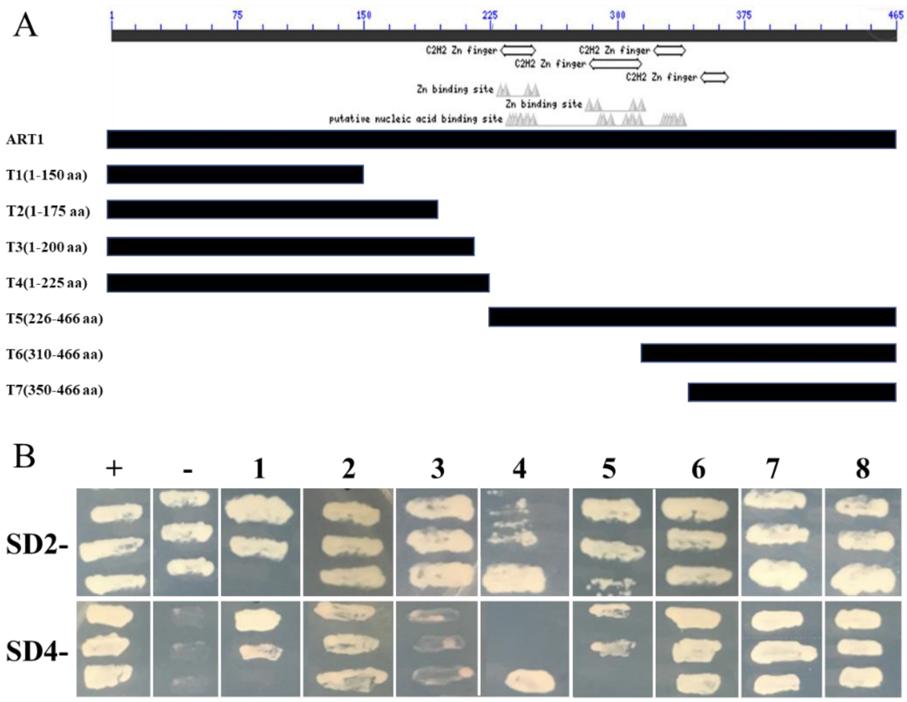

Figure S1. Transcriptional activation analysis of various truncated ART1s. (A) ART1 was truncated into different fragments, including T1 (1-150 aa), T2 (1-175 aa), T3 (1-200 aa), T4 (1-225 aa), T5 (226-466 aa), T6 (310-466 aa) and T7 (350-466 aa). (B) Identification of self-activation domain of ART**. +** : AD-T7 + BK-53, **-** : AD + BK, 1: ART1-BK + AD, 2: ART1-T1-BK + AD, 3: ART1-T2-BK + AD, 4: ART1-T3-BK + AD, 5: ART1-T4-BK + AD, 6: ART1-T5-BK + AD, 7: ART1-T6-BK + AD, 8: ART1-T7-BK + AD, the above pair of plasmids were co-transformed into yeast cells(AH109). The yeast colonies were grown on SD2- and SD4- medium plate. The plates were incubated at 30 °C for 3 d.

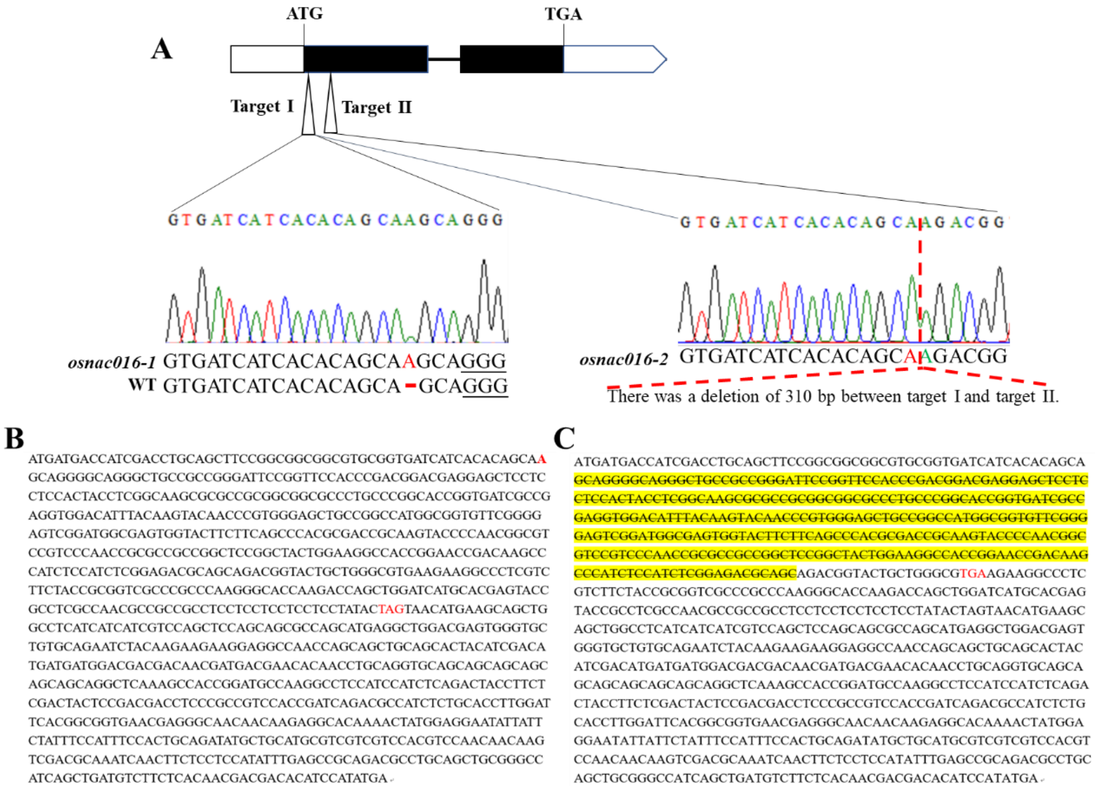

Figure S2. Mutated sequence of two independent *OsNAC016*-knockout lines generated by CRISPR/Cas9 mutagenesis. (A) Black box indicates the exon. The *osnac016-1* mutant has a base A insertion at the position of target one. The *osnac016-2* mutant had a 307 bp deletion between target I and target II. (B) Open reading frame analysis of mutated *OsNAC016* in the *osnac016-1* line. (C) Open reading frame analysis of mutated *OsNAC016* in the *osnac016-2* line. a premature stop codon was produced at 502 bp in the o*snac016-1* line and at 386 bp in the o*snac016-2* line, respectively.

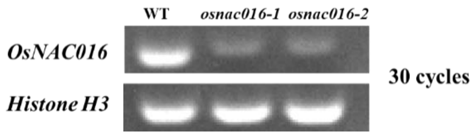

Figure S3. Transcript level of *OsNAC016* in roots of the wild-type and *osnac016* plants. The expression level of *OsNAC016* in the roots of 5-d-old rice seedlings were determined by semi-quantitative RT-PCR. *Histone H3* was used as an internal standard.

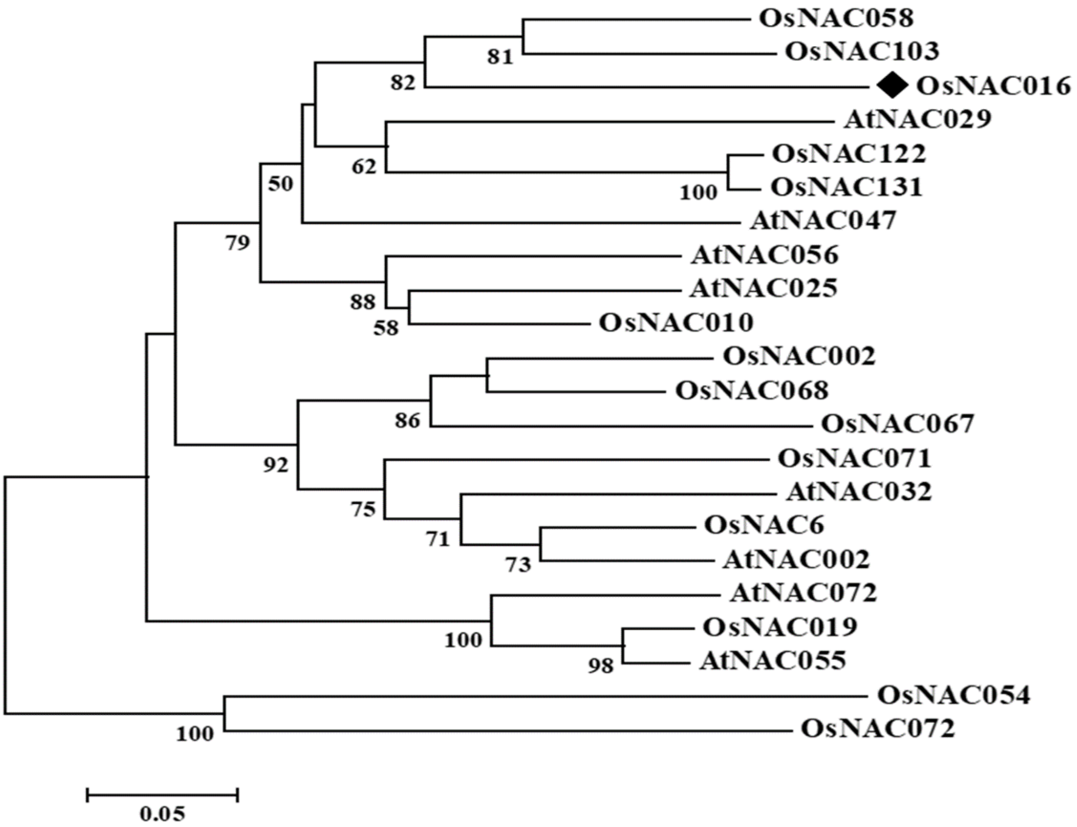

Figure S4. Phylogenetic tree analysis of *OsNAC016*. Homologs sharing with more than 40% identity to OsNAC016 were used to construct the phylogenetic tree. Multiple sequence alignment was performed using the MEGA 6.0 and the phylogenic tree was created.

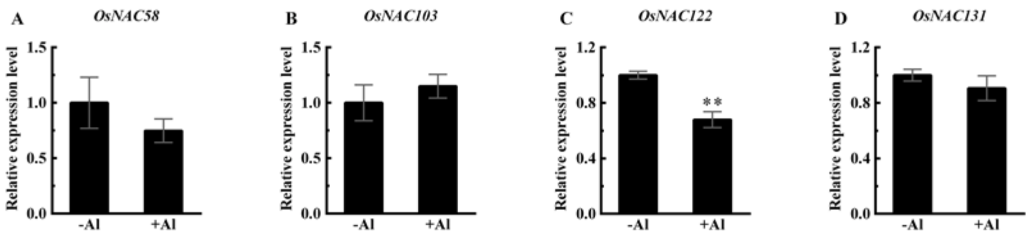

Figure S5. Expression analysis of four OsNAC016 homologous genes in roots. (A-D) Rice seedlings (5-d-old) were subjected to a solution containing 0 or 30 μM $AlCl_3$ for 8 h. Histone H3 was used as an internal standard. Data are means ± SD (n = 3). Asterisks indicates significant differences ($P < 0.01$, Tukey's test).

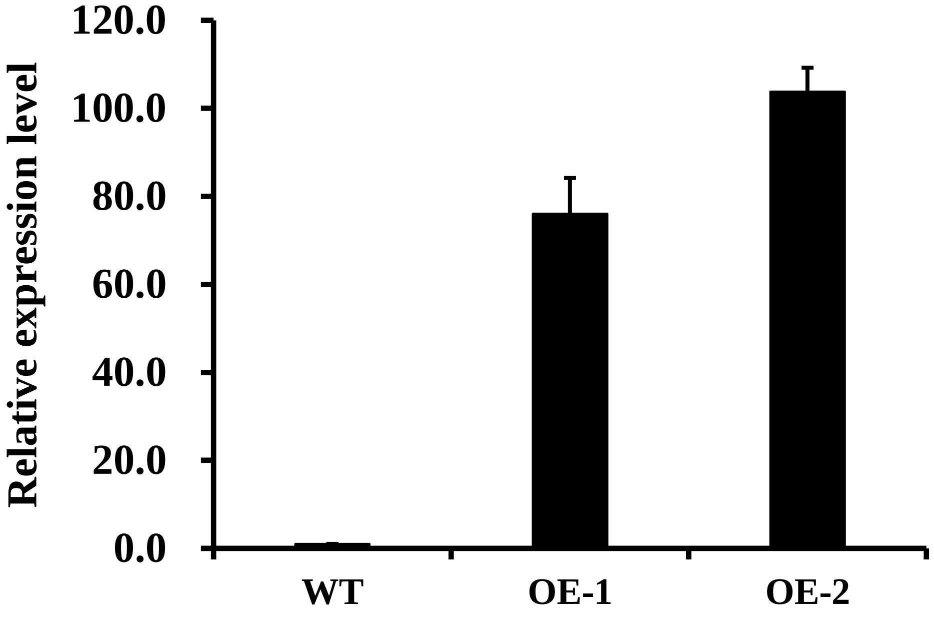

Figure S6. Relative expression levels of *OsNAC016* in the *OsNAC016*-overexpression lines (OE-1, OE-2) in roots. Data are means ± SD of three biological replicates.

ATCTAATCATTAATTGTCAAAAGACATTGAGCACATCATCCTCCCTCGTTGCTGCACATACAATT
GAGATATTTTATTTTCTTCAACTCTAAACTACAATTTTCTCATTTTCCGTTAGCACACTATACAAA
TTGTTAAATAGTATTATGTTTTTTTTTTCAAAAACTGTTTATATATAAATTTCTTTAAATAAATTTA
TCTTTAAAGTTTATTATAATTAATTATATGCTAATGTCTTATCTCACTTTATGTGTCGTTGAAAAG
CTAAACCAAATTGTGCTGATTTGAATTAAGCCCGAGTCACTCGCATATGAGTAGTCCTATTTGTT
ACGGTCTCACATGCGAATGATTTAGCTTCCTTATACTATACTCTTTTCGTAACAAAATATAATAA
CCTACTACTGGAACATAAAACCTGTTTAGATTCTTAATACTTGACGGAGCGTGGGGCTCCTCAC
CGGGAGACCGCGCGGGCCCCCCTTTGCCGGTTCGGCCGGGGGCGCTAAGTGAGGTTCTAAGCC
CTTGATCTGTGGAATGGTTCGCGAGAGAGTAAATGCACAAGACACGGGCGATGTAGACAGGTT
CGGGCCGCTGAGAAGCGTAATACCCTACTCCTGTGTTCTGGTGGATCTGTGTATGAAGGAGTTA
CAAAGAGCTGGAGAGCAAGAGAGTTCGAACTCTAGAGACCCTCTCCCTACTCTTTTTCCCAGTC
CCCTCTTTTTCTTTTCCCCCCGTCCGTCAGTCCGTCAGTCCCCCCCTCTTGGTAGGCATGGTCCTC
CTTTTATATCTCAAGGGGATACCACATACACCATTTCTACCTACCCTTCTTTTGGGTGGGGACCC
ATCCTACTTTGACCAAAACTTGGCTTGTACCTTCGTCTGACACCGGGATAACCCTGTCCTCCCTC
ATTAATGGGCGGAGATTTGCAATGGCTGCCGTCCGAATGGCCCTCCGATGGGATGGGTCATACC
AACCTCCACTCCGCCGGAAGCAGGTGCAACGTGGAAGCACGGTTGTTTGCTGATGACGCGACC
GGCGTCAGACCAGTCACAGACCGGTCATTCTTGTCCACCACGTGTCAGTTTAGCATGCTACACG
TCTGCCCTTCTTCATACAATGACTTCCTTGCAATGGTTGCGATGAAGCCTGGAATGTATCTAGCC
GGGGCTGACGTGCCAACTCCAGGAGTCCCCACGCCGGCTCCGGCTAGGGACGAGTGCCTGGAG
GCTCTCATCATTCCGACGGGACGGGGCGAGGCGTGTGTCAGGCCGCCTGTTGCCCCCTAACCCG
CGATCTGACCGGTCTGTGACCGGTCACACACTGCGACCGATCACGGACTAGATAAGCATGTGCT
ACGCTGCATTAAATGCGGCGTGAGACGCTCGTCCAACCCGCAATAAACGCGGTTAGGTGAGCG
CCGCTGTGCTCATCTAACCCACACACGTGGCGCCAAACCCTCAGGGGGTCGGGGCGCCCCAGC
CCTCGGGGACGAAACGGGAGCGGCCCGACCCCTCGGGGAGATAGAGGGAGGGGCGGCCTCAT
CACCCTCGGGCCCGACCCCCCCGAGGGGGTCAGGCCACGTGGGTGACCGCGGCTGCCCCAAAC
CTCTAGTCAAGATATCCCCGGTCCCATGTCACCGACAATACTAATAAGATAAATCTTATCCGAC
ACGGAAAGAGATCCTCTTACCTACTTGTTGTGCAGTTGAATTACTGATATATAAACCACAACAA
AGTAGGATTAATTCATATATCAGTGACAACTACACAGTTAGGTCAAAGGATCCGCTTCCATCCG
GCACTAGGTTGGGAGTATTACCATACAAGCTTCCCTTGCTGCCTGCTGCTGGCCACTCCTTGTGT
CCACTGTCCACAGATACAAACTCCATTCGTAATTTCGTACGGCCGGTCGGGTTACATCTAGTGC
AGGCACGAACACGTACAGACTCCCCTGATTTTTGGATCTATTTAAGCCGAGCTGAGTGAACCAA
TTGGCCACC

Figure S7: Cis-acting element analysis of *OsFRDL4* promoter.

NAC TFs of cis-acting element: CACG.

ART1 TF of cis-acting element: [GGN(T/g/a/C)V(C/A/g)S(C/G)].
